# Sinusless Chronic Dento-Alveolar Abscess (Dental Granuloma)

**Published:** 1918-07

**Authors:** Julio Endelman


### SINUSLESS CHRONIC DENTO-ALVEOLAR ABSCESS (DENTAL GRANULOMA)

BY JULIO ENDELMAN, D.D.S.

### PRELIMINARY NOTE

Notwithstanding the fact that dental practitioners, and in particular dental investigators of problems in the field of pathology and therapeutics, have been devoting much time and attention to the character of the lesion in the cementum, peridental membrane, alveolar process and in the alveolar bone, still the character of the pathologic reaction which follows the invasion of the periapical peridental membrane by bacteria of low virulence has not as yet been definitely established. As the result of this unsatisfactory conditions of affairs, two different schools of thought in connection with this problem have developed, viz., the school of radicalism, and the school of conservatism. The radicals assume that a chronic periapical infection with its reaction in the shape of a dental granuloma, is incurable by any and all means available, and resort to extraction. The conservatives argue that from the standpoint of clinical results all teeth whose roots are the seat of chronic periapical infections are not beyond the possibility of successful treatment, and consequently aim to eradicate the infection. They claim that frequently success accompanies their efforts.

As a result of the writer's investigation of the pathology of these lesions, which has been conducted for some time past, he is now led to conclude that both views of the question are correct, each as depicting a different pathologic reaction. Authors of the few textbooks and writers on the subject of the pathology of chronic peridental infections (sinusless chronic dental granuloma), have invariably asserted that as the result of the infection of the peridental membrane (which leads to the reaction under consideration—dental granuloma), the peridental membrane in the periapical region is detached from the cementum; that as the result of this detachment the cementoblasts in the lacunae of the cementum die; and finally, that the area of cementum thus deprived of peridental membrane, and cementoblasts in the lacunae, undergoes necrosis. It is the purpose of this preliminary note to state that detachment of the peridental membrane, and consequent death (necrosis) of the cementum, do not occur as a constant accompaniment of chronic infections of the periapical peridental membrane. The detachment or destruction of the peridental membrane in this location is the advanced stage in the process, and occurs only as the result of an exacerbation of the infection. In other words, when the dental granuloma breaks down, and the inflammatory cells which make up the bulk of the granuloma begin to undergo a process of liquefaction, then in time this affects the periapical peridental membrane. A sinusless chronic dento-alveolar abscess (dental granuloma) which shows in a radiograph as a dark area in the neighborhood of the apex of the root is not an evidence that the apex of the root is in a necrosed state. The lesion, until such time as the infection acquires increased virulence, does not lead to tissue destruction, but to tissue proliferation in the peridental membrane. The infection appears to lead to tissue stimulation rather than to tissue liquefaction, and the formation of the fibrous wall is a bit of evidence in support of this contention. Until such times as the degree of virulence of the bacteria concerned in periapical infections changes from cronicity to subacuteness or acuteness the lesion is destructive in the surrounding alveolar bone and constructive (tissue proliferation) in the peridental membrane.—*Pacific Dental Gazette.*

